# Neutrophil percentage-to-albumin ratio as predictor for mortality in patients undergoing endoscopic intervention for variceal hemorrhage

**DOI:** 10.1186/s40001-025-02489-4

**Published:** 2025-04-26

**Authors:** Nasser Mousa, Sherif Elbaz, Amr Elhammady, Mostafa Abdelsalam, Eman Abdelkader, Mohamed Wahba, Niveen El-wakeel, Ola El-Emam, Wesam Elderiny, Ayman Elgamal, Alaa Elmetwalli, Ali El-Assmy, Mohammed Abdelaziz, Abdel-Naser Gadallah, Marwa Mansour

**Affiliations:** 1 Tropical Medicine Department, Mansoura University, Mansoura, Egypt; 2 Egyptian Liver Research Institute and Hospital (ELRIAH), Mansoura, Egypt; 3 Endemic Diseases and Gastroenterology Department, Aswan University, Aswan, Egypt; 4 Nephrology fellow, HCA Houston Healthcare Kingwood/University of Houston , Houston- Texas , USA; 5 Internal Medicine Department, Banha University, Banha, Egypt; 6 Mansoura Nephrology and Dialysis Unit, Mansoura University, Mansoura, Egypt; 7 Alameen General Hospital, Taif, Kingdom of Saudi Arabia; 8 Internal Medicine Department, Mansoura University, Mansoura, Egypt; 9 Medical Microbiology and Immunology Department, Mansoura National University, Mansoura, Egypt; 10 Medical Microbiology and Immunology Department, Faculty of Medicine, Delta University for Science and Technology, Gamasa, Egypt; 11 Clinical Pathology Department, Mansoura University, Mansoura, Egypt; 12 Tropical Medicine Department, Menoufia University, Menoufia, Egypt; 13 Prince Fahad bin Sultan Research Chair for Biomedical Research, University of Tabuk, Tabuk, Saudi Arabia; 14 Medical Student in Faculty of Medicine Mansoura University, Mansoura, Egypt; 15 Internal Medicine Department, Menoufia University, Menoufia, Egypt

**Keywords:** Neutrophil percentage-to-albumin ratio, Variceal bleeding, Mortality, Liver cirrhosis, Portal hypertension

## Abstract

**Background:**

Variceal bleeding (VB) is a serious condition that can lead to increased hospital costs and mortality rates. The neutrophil percentage-to-albumin ratio (NPAR) has been recognized as a predictor of mortality in various diseases. However, the use of NPAR as a predictor of hospital mortality in patients with VB has not been studied previously.

**Aim:**

To assess the effectiveness of NPAR in predicting mortality in VB patients undergoing endoscopic treatment**.**

**Methods:**

This study included 415 cirrhotic patients who were hospitalized for an upper gastrointestinal bleeding and had endoscopy. NPAR was computed at index admission in blood samples. Using the receiver operator characteristic curve (ROC), the sensitivity and specificity of the NPAR for predicting mortality in patients with VB were calculated.

**Results:**

Out of 415 cirrhotic patients, 322 patients with variceal bleeding as the sole culprit bleeding lesion were included in the study, while 93 patients with different bleeding lesions were excluded. Among the 322 patients included in the study, 29 (9%) patients died in hospital. The predictors of death for the cases were NPAR (*p* = 0.0001, AOR: 1.11, 95% CI 1.06–1.16), hospital stay (*p* = 0.006, AOR: 1.39, 95% CI 1.10–1.76), and pulse rate (*p* = 0.0001, AOR: 0.936, 95% CI 0.907–0.965). ROC analyses showed that NPAR at a cut-off value of 27.8 had optimal discriminative power for differentiating between alive and deceased cases with a sensitivity of 82.8%, specificity of 65.9%, PPV of 19.4%, NPV of 97.5%, and an accuracy of 67.4% (*p* < 0.001).

**Conclusions:**

NPAR may be a useful predictor of mortality in patients with VB.

## Introduction

The Neutrophil Percentage-to-Albumin Ratio (NPAR) is a novel inflammatory biomarker that integrates neutrophil and albumin levels. Neutrophils, the main white blood cells that combat bacterial infections, are essential for evaluating infection severity and determining the necessity for intensive care unit admission [1]. Albumin, a crucial protein in the body, plays a vital role in osmoregulation, antioxidation, and anti-inflammation. Alterations in the structure and function of albumin are linked to conditions such as cirrhosis [2]. NPAR is a cost-effective tool for predicting mortality in various patient groups, including cardiovascular death in diabetes, cerebrovascular mortality in hypertension, advanced liver fibrosis in MASLD patients, mortality in chronic kidney disease, and infection in cirrhosis patients [3–8].

Portal hypertension is the primary cause of complications in patients with cirrhosis, leading to issues such as gastrointestinal bleeding, hepatic encephalopathy, spontaneous bacterial peritonitis, and ascites [9–14]. Esophageal varices are present in approximately half of individuals newly diagnosed with liver cirrhosis. Among those with varices, up to 25% will experience variceal bleeding, which carries a high mortality rate of 25–50% and significant healthcare costs despite advancements in diagnosis and treatment. The mortality associated with variceal bleeding is substantial, accounting for 13–19% of overall mortality in cirrhosis [15]. Several risk scoring systems have been developed to assess the prognosis of upper gastrointestinal bleeding (UGIB), such as the Glasgow-Blatchford, Child–Pugh score (CPS), AIMS65, and Rockall scores [16].

These ratings are inadequate for predicting adverse outcomes in patients with variceal hemorrhage, as some of the data used in these scores is subjective and not specific to VB [17, 18]. Additionally, integrating this prognostic information into daily clinical practice may pose challenges for clinicians [19].

The aim of this study is to evaluate NPAR as a non-invasive indicator for in-hospital mortality in cirrhotic patients with variceal bleeding.

### Patients and methods

This prospective observational study involved 415 cirrhotic individuals who were admitted with an episode of UGIB to the Tropical Medicine Department at Mansoura University, the Internal Medicine Departments at Mansoura, Menoufia, and Banha Universities in Egypt, and the Egyptian Liver Research Institute and Hospital (ELRIAH). In-hospital mortality was defined as death that occurred during the hospitalization for the specific variceal bleeding episode.

#### Inclusion criteria

Cirrhotic patients aged 18 years or older presented with documented UGIB. UGIB was defined as hematemesis (fresh blood, coffee grounds emesis) and melena (passage of black, tarry stools) in the setting of an established upper gastrointestinal tract culprit bleeding lesion. Cirrhosis was diagnosed based on history, clinical, laboratory, and radiographic tools.

#### Exclusion criteria

Participants under 18 years of age, those with bleeding from sources other than varices (such as peptic ulcers or portal gastropathy), individuals who withdrew their informed consent, and those who refused endoscopy were excluded from the study. Patients with serious diseases affecting other organs that could impact survival, such as heart failure, coma, recent myocardial infarction, cardiopulmonary instability, hypertensive shock unresponsive to initial resuscitation, and fever exceeding 37.5 °C during the index admission, were not included. Additionally, individuals with infections in other sites affecting neutrophil count, such as pneumonia evident on chest x-rays or other infections diagnosed based on clinical, radiological, and bacteriological data, were also excluded.

Upon admission, all patients underwent a comprehensive assessment, which included a detailed medical history, physical examination (including vital signs such as blood pressure, heart rate, respiratory rate, and body temperature), and radiological imaging. Laboratory tests were conducted to assess liver function (ALT, AST, albumin, bilirubin, and prothrombin time), creatinine levels, complete blood count (neutrophil percentage determined using the CELL-DYN Emerald cell counter from Abbott in Wiesbaden, Germany), international normalized ratio (INR), and radiographic examinations. The NPAR was calculated by dividing the neutrophil percentage (numerator) by the albumin level (denominator) in the same blood samples collected upon admission [20].

All patients received proton pump inhibitor infusions upon admission, along with standard conservative care including blood transfusions as needed, vasoactive drugs, and ceftriaxone 1 g/24 h for 5 days. Endoscopy was performed within the first 8–12 h under midazolam sedation, and endoscopic treatment was carried out by equally experienced and licensed endoscopists. The primary diagnosis was identified as a bleeding lesion, with varices considered to have hemorrhaged if they exhibited bleeding stigmata. The primary endpoint was to assess whether the NPAR measurement could predict mortality in cases of UGIB.

### Statistical analysis

All patients received proton pump inhibitor infusions upon admission, along with standard conservative care including blood transfusions as needed, vasoactive drugs, and ceftriaxone 1 g/24 h for 5 days. Endoscopy was performed within the first 8–12 h under midazolam sedation, and endoscopic treatment was carried out by equally experienced and licensed endoscopists. The primary diagnosis was identified as a bleeding lesion, with varices considered to have hemorrhaged if they exhibited bleeding stigmata. The primary endpoint was to assess whether the NPAR measurement could predict mortality in cases of UGIB.

## Results

**Fig. 1:**
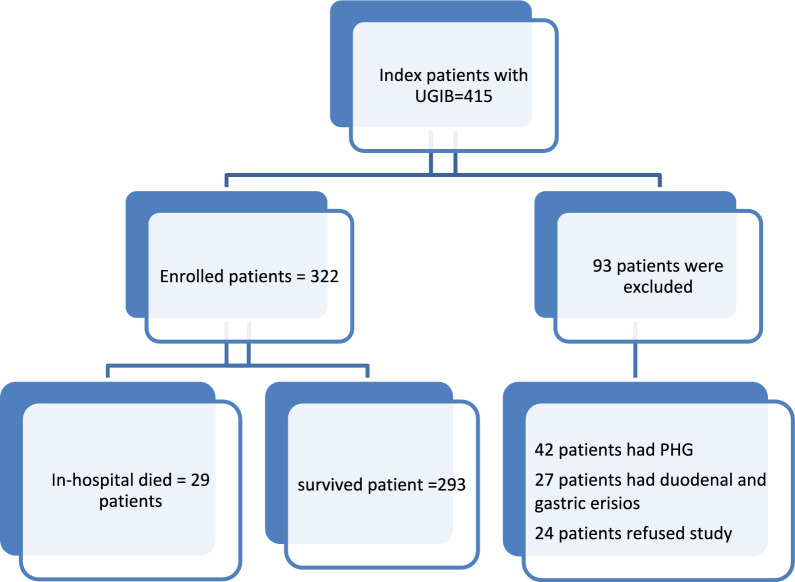
Follow chart of study patients

All patients received proton pump inhibitor infusions upon admission, along with standard conservative care including blood transfusions as needed, vasoactive drugs, and ceftriaxone 1 g/24 h for 5 days. Endoscopy was performed within the first 8–12 h under midazolam sedation, and endoscopic treatment was carried out by equally experienced and licensed endoscopists. The primary diagnosis was identified as a bleeding lesion, with varices considered to have hemorrhaged if they exhibited bleeding stigmata. The primary endpoint was to assess whether the NPAR measurement could predict mortality in cases of UGIB (Fig. 1).

Variables for instances who are alive and those who have died are compared in (Table 1). Patients who died showed a significantly higher prevalence of ischemic heart disease, chronic renal illness, bradycardia, and lower blood pressure (both systolic and diastolic) than patients who survived an esophageal bleeding incident. Additionally, compared to patients who are still alive, those who passed away had a significantly lower serum albumin, hemoglobin level, and platelet count. However, they also had a significantly higher total leukocyte count, neutrophil percentage, INR ratio, serum creatinine, bilirubin, and neutrophil-to-albumin ratio, as well as a longer hospital stay.

**Table 1:** Comparison of baseline demographic and laboratory data at index admission between alive and died cases

	Total *N* = 322	Alive *N* = 293	Death *N* = 29	*P* value
Age/years	58.16 ± 13.48	57.95 ± 13.89	60.31 ± 8.17	0.37
Sex				
Male	224	202 (90.2)	22 (9.8)	0.44
Female	98	91 (92.9)	7 (7.1)	
Co-morbidities				
Diabetes Mellitus	127	111 (87.40%)	16 (12.5%)	0.69
Hypertension	84	79 (90.00%)	5 (10.00%)	0.26
Ischemic heart disease	30	23 (7.80%)	7 (24.13%)	0.04
Chronic kidney disease	18	14 (4.77%)	4 (13.00%)	0.04
Pulse/min	88.77 ± 13.61	89.71 ± 13.04	79.31 ± 15.75	< 0.001
Systolic blood pressure	101.83 ± 13.68	102.56 ± 12.60	94.48 ± 20.63	0.002
Diastolic blood pressure	65.11 ± 9.82	65.71 ± 9.17	58.92 ± 13.70	< 0.001
Temperature	37.70 ± 0.31	37.50 ± 0.31	37.90 ± 0.37	0.89
Respiratory rate	18.95 ± 1.01	18.94 ± 1.01	19.10 ± 1.01	0.39
TLC (10^3^/cm^3^)	11.8 (9.2–15.35)	11.7 (8.9–14.50)	16 (10.8–22.80)	0.002
Neutrophil %	75.27 ± 7.32	74.74 ± 7.12	80.65 ± 7.32	0.001
Hemoglobin (g/dl)	8.1 (6.90–9.23)	8.20 (6.95–9.30)	7.10 (5.80–9.05)	0.012
Platelets (10^9^/L)	111 (78–199.25)	111 (78–201)	104 (76–172.5)	0.57
INR (Ratio)	1.62 (1.35–1.81)	1.6 (1.34–1.80)	1.99 (1.73–2.80)	< 0.001
Serum creatinine (g/dL)	1.23 (1.10–1.50)	1.21 (1.05–1.35)	1.86 (1.46–3.10)	< 0.001
Serum albumin (g/dL)	2.91 ± 0.59	2.96 ± 0.58	2.39 ± 0.58	< 0.001
Serum bilirubin(mg/dL)	1.53 (1.20–2.40)	1.41( 1.20–2.20)	3.8 (1.58–8.60)	< 0.001
NPAR	26.15 (22.66–30.37)	25.71 (22.23–29.49)	35.20 (29.25–40.50)	< 0.001
Blood transfusion/U	2.0 (1.0–2.0)	2 (1–2)	2 (1–4)	0.70
Hospital stay (days)	4 (3–4)	2 (1–2)	5 (4–6)	< 0.001

*TLC* total leucocyte count, *NPAR* Neutrophil count to albumin ratio

The death predictors for the cases under study are displayed in (Table 2). The death rate of patients was found to be statistically significantly correlated with NPAL (*p* = 0.0001, AOR: 1.11, 95% CI 1.06–1.16), pulse rate (*p* = 0.0001, AOR: 0.936, 95% CI 0.907–0.965), INR (*p* = 0.007, AOR: 2.762, 95% CI 1.31–5.81), and hospital stay (*p* = 0.006, AOR: 1.39, 95% CI 1.10–1.76).

**Table 2:** Predictors of mortality among studied cases

	β	AOR (95% CI)	*P* value
Ischemic heart disease	0.99	2.693 (0.92–7.82)	0.07
Chronic kidney disease	1.006	2.735 (0.71–10.46)	0.147
Pulse/min	− 0.066	0.936 (0.91–0.96)	0.001*
Systolic blood pressure	− 0.005	0.995 (0.92–1.08)	0.89
Diastolic blood pressure	− 0.067	0.935 (0.84–1.05)	0.25
TLC/(10^3^/cmm)	− 0.006	0.994 (0.94–1.05)	0.84
Neutrophil %	0.056	1.058 (0.97–1.15)	0.20
Hemoglobin (g/dl)	− 0.070	0.932 (0.69–1.26)	0.65
Platelets (10^9^/L)	0.004	1.00 (0.99–1.01)	0.20
INR	1.016	2.762 (1.31–5.81)	0.007*
Serum creatinine (g/dl)	0.163	1.177 (0.92–1.51)	0.21
Serum albumin (g/dL)	− 1.19	0.305 (0.12–0.77)	0.012
Serum bilirubin(mg/dL)	− 0.38	0.685 (0.27–1.72)	0.42
Neutrophils/Albumin ratio	0.104	1.11 (1.06–1.16)	< 0.001*
Hospital stay (days)	0.33	1.39 (1.10–1.76)	0.006*
Overall % predicted = 90.4%			

*AOR* adjusted odds ratio, *TLC* total leucocyte count

*Significant

Table 3 displays the correlation between the analyzed patients' study characteristics and NPAR. Age, total leukocyte count, neutrophil percentage, INR ratio, serum creatinine, bilirubin, and hospital stay were all significantly positively correlated with NPAR, whereas hemoglobin level, platelet count, and albumin were significantly negatively correlated.

**Table 3:** Correlation between Neutrophils to Albumin ratio and analyzed patients' study data

	Neutrophils/Albumin ratio	
	*r*	*P* value
Age/years	0.21	< 0.001
Pulse	0.05	0.33
Systolic blood pressure	0.04	0.53
Diastolic blood pressure	− 0.02	0.70
Temperature	0. 08	0.89
Respiratory rate	− 0.06	0.27
TLC (10^3^/cmm)	0.48	< 0.001
Neutrophil %	0.62	< 0.001
Hemoglobin (g/dl)	− 0.29	< 0.001
Platelets (109/L)	− 0.38	< 0.001
INR	0.48	< 0.001
Serum creatinine (g/dl)	0.41	< 0.001
Serum albumin (g/dL)	− 0.89	< 0.001
Serum bilirubin(mg/dL)	0.48	< 0.001
Blood transfusion/units	0.07	0.40
Hospital stay (days)	0.26	< 0.001

*r* Spearmancorrelationcoefficient

**Fig. 2:**
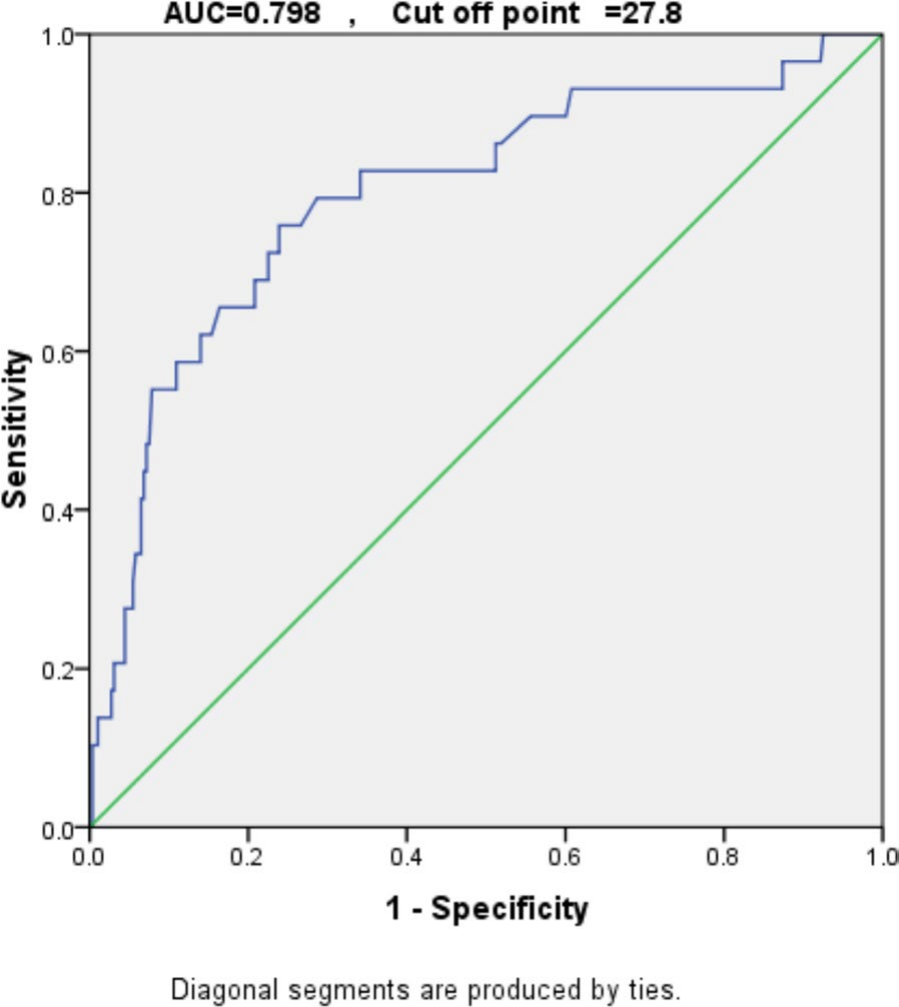
ROC curve of Neutrophils/Albumin ratio in differentiating between alive and died cases

**Fig. 3:**
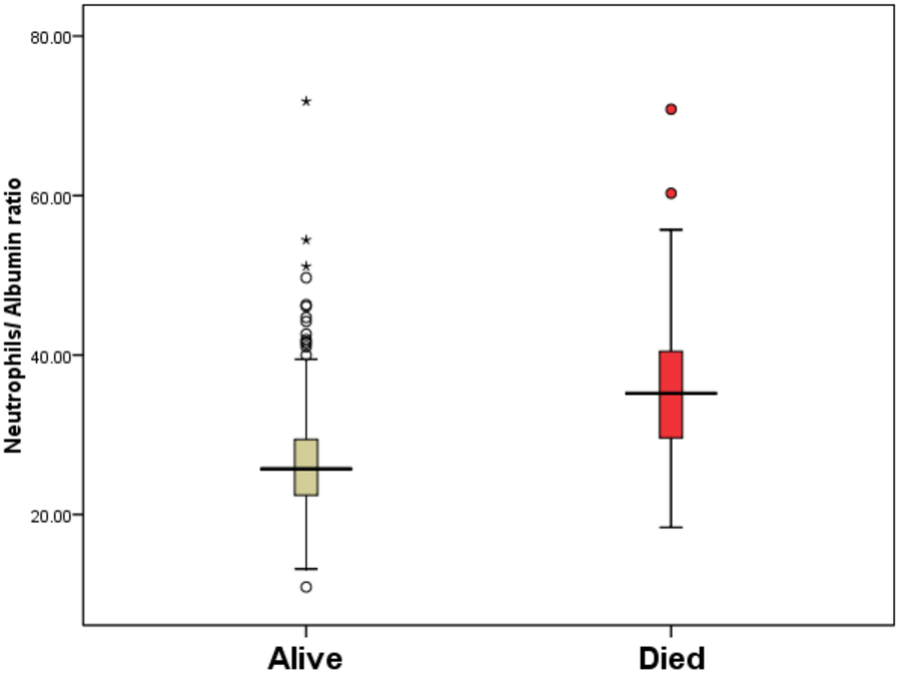
Box and Whisker plot showing median Neutrophils/Albumin ratio in differentiating between alive and died cases

Table 4, Figs. 2 and 3 show the validity of NPAR in differentiating outcomes. The ROC curve analysis demonstrated that at a cut-off value of 27.80, NPAR had optimum discriminative power for differentiating between alive and deceased cases, with a sensitivity of 82.8%, specificity of 65.9%, PPV of 19.4%, NPV of 97.5%, and accuracy of 67.4% (*p* < 0.001).

**Table 4:** Validity of Neutrophils/Albumin ratio in differentiating outcome

Neutrophils/Albumin ratio	AUC (95%CI)	*P* value	Cut off point	Sensitivity %	Specificity %	PPV%	NPV%	Accuracy %
Differentiating between alive and died cases	0.798 (0.705–0.892)	< 0.001*	27.80	82.80	65.9	19.40	97.50	67.40

*AUC*, area under curve, *PPV* positive predictive value, *NPV* negativepredictive value

*Significant

## Discussion

This study aims to investigate the diagnostic utility of NPAR as a predictor of death in patients presenting with VH. The most frequent cause of gastrointestinal bleeding in cirrhosis patients is variceal hemorrhage, accounting for 70% of cases with high mortality rate [9, 11]. Many factors, such as high MELD scores, kidney failure, hepatic venous pressure gradients above 20 mm Hg, and endoscopic evidence of an ongoing bleed, are associated with higher mortality and a worse prognosis [21–23]. Numerous studies have demonstrated the good predictive value of NPAR in patients with a variety of diseases [12, 13].

The utility of NPAR as a predictor of death in cirrhotic patients with bleeding varices can be explained in at least two ways. Firstly, since infection is a common cause of death during variceal bleeding and is frequently seen in advanced cirrhotic patients, it is crucial to consider the role of neutrophils in reflecting immune dysregulation and systemic inflammation. Moreover, infection has been associated with unfavorable outcomes and a high mortality rate. Secondly, hypoalbuminemia, due to inflammation and malnutrition, has been demonstrated to be a dependable predictor of a poor prognosis in cirrhosis [24, 25].

Our study revealed that the NPAR was significantly higher in individuals who died compared to those who survived the incident of esophageal bleeding. Furthermore, the NPAR is a significant and independent predictor of in-hospital mortality in patients with variceal bleeding. Through multivariate logistic regression analysis, NPAR consistently showed a strong significant association with mortality (AOR: 1.11, 95% CI 1.06–1.16, *p* < 0.001), even after adjusting for other significant factors such as hospital stay, INR, and pulse rate. NPAR uniquely integrates inflammatory and nutritional status, making it a more holistic and practical predictor [4, 26]. These results suggest that NPAR could serve as a marker for stratifying mortality risk in clinical practice, particularly in resource-limited settings where rapid and cost-effective assessments are crucial. By consolidating these findings, we emphasize NPAR's potential as an independent and practical tool for predicting mortality in this high-risk population.

We calculated the discriminative power of NPAR to distinguish between survivors and non-survivors using Receiver Operating Characteristic (ROC) curve analysis. For our study, NPAR yielded an AUC of 0.798, which reflects good discriminative power. The optimal cutoff value of 27.8 was determined using Youden's index, which balances sensitivity and specificity. At this cutoff, NPAR achieved a sensitivity of 82.8% and a specificity of 65.9%, indicating its ability to reliably differentiate between patients at high and low risk of mortality. The strong discriminative power of NPAR, as evidenced by the ROC curve and its associated metrics, supports its potential as a valuable prognostic tool in clinical practice. This approach aligns with established statistical methodologies for evaluating diagnostic and prognostic markers. This is consistent with Du et al., who identified NPAR as a predictor of mortality risk in patients with decompensated cirrhosis. They demonstrated that a higher risk of death in patients with liver cirrhosis was independently correlated with an increased NPAR, with a sensitivity of 63.72% and specificity of 59.08% [26]. Han et al. also found that ROC curve analysis used to determine the utility of NPAR for predicting death had a sensitivity of 68.8% and specificity of 73.9% in their investigation into the usefulness of NPAR in predicting the outcomes of patients with decompensated cirrhosis [27].

Albumin has multiple functions including osmoregulation, anti-oxidation, and anti-inflammatory properties. It is used to predict mortality in critically ill cirrhotic patients [28]. Cirrhosis not only reduces albumin synthesis but also affects its structure and function. Decreased serum albumin levels are associated with the severity of liver cirrhosis, and hypoalbuminemia is a strong predictor of poor prognosis in cirrhosis due to inflammation and malnutrition [24, 26]. In the present study, serum albumin level was significantly lower in dead patients versus survivor's patients and had a significant negative correlation with NPAR. Since albumin is the denominator in NPAR, a decrease in it is linked to an increase in this ratio in hospitalized deaths. This finding could be explained by the presence of an inverse correlation between systemic inflammatory response and serum albumin levels. Furthermore, survival probability decreases by 63.4% when serum albumin is ≤ 2.45 g/dl [29].

Neutrophils are a crucial indicator of inflammation and immune system imbalance. Studies show that elevated neutrophil levels can predict a negative outcome in patients with VB [1]. The current research reveals that non-survivors had significantly higher neutrophil counts compared to survivors. Neutrophil dysfunction is commonly linked to cirrhosis, suggesting that increased circulation of these cells, particularly the proinflammatory subset, may indicate a poor prognosis [30]. Additionally, underlying infections or inflammation may have contributed to the patients' fatalities.

NPLR has been shown to predict outcomes in chronic liver disease by affecting pathways related to low-dose endotoxinemia, leading to systemic inflammation in cirrhosis patients [31, 32]. Additionally, cirrhosis patients may have neutrophil functional defects, increasing their risk of organ failure, infection, and mortality [33]. Also, neutrophils play a crucial role in the initial inflammatory response to acute infections. A high neutrophil count can indicate a systemic infection. Neutrophils are recruited during acute-phase reactions and can be used to evaluate disease severity and tissue inflammation [34].

This study found a significant negative correlation between hemoglobin levels, platelet count, and serum albumin. The decreased platelet count suggests advanced portal hypertension, splenic sequestration, and reduced liver thrombopoietin synthesis [35]. These factors indicate the severity of liver dysfunction and are associated with poorer clinical outcomes. Conversely, an elevated NPAR is linked to systemic inflammation (elevated neutrophil percentages) and hypoalbuminemia which is also hallmarks of advanced liver disease and poor prognosis. This negative correlation may indicate a meeting of hemostatic dysfunction and systemic inflammation and nutritional depletion, and in advanced disease. The association of decreasing platelet counts with elevated NPAR may indicate the risk of adverse outcomes. These findings highlight the complicated relationship between inflammatory and hemostatic markers in cirrhosis, suggesting NPAR as a comprehensive marker of disease severity. Previous studies support these findings, showing that higher NPAR levels are associated with more severe liver dysfunction, cirrhosis-related complications, and an increased risk of mortality [26, 36].

This study found a strong positive correlation between NPAR and elevated serum bilirubin levels. Previous studies by Du et al. [26] and Han et al. [27], also reported similar associations between NPAR and increased serum bilirubin levels in cirrhotic patients. Additionally, our study showed a significant positive correlation between NPAR and hospitalization duration. These results support existing evidence of a connection between high NPAR and longer hospital stays [26, 27].

Our study found that NPAR is a significant and independent predictor of in-hospital mortality in cirrhotic patients with variceal bleeding. Even after adjusting for established mortality predictors like hospital stay, INR, and pulse rate, NPAR remained significant in multivariate logistic regression analysis. This highlights the value of NPAR as a prognostic marker. However, it is important to note that while NPAR is a strong predictor, other factors may also influence mortality risk. Factors like INR, serum albumin, and MELD scores are crucial in assessing mortality risk [21, 22, 37, 38]. In this study, a significant positive correlation between NPAR and INR was identified. Elevated INR is linked to a negative prognosis in patients with liver failure and is a predictor of severe esophageal varices. This association may be attributed to the compromised liver function, leading to impaired synthesis of coagulation factors and albumin [39].

Further research is needed to compare the importance of NPAR relative to these factors. NPAR's simplicity and ease of measurement make it a practical and cost-effective tool for risk assessment, but it should be used alongside other clinical and laboratory parameters. Future studies should validate our findings and explore how NPAR fits into broader predictive models or existing scoring systems to validate its role in clinical decision-making.

## Conclusion

NPAR is inexpensive, objective, and simple to use. The NPLR's strong correlation with death in VB supports the use of this measure as a prognostic indicator, according to our findings.

## Data Availability

The data used in this study is available upon a reasonable request to the corresponding author, and after permission of all participating services.
